# Portable eye-tracking as a reliable assessment of oculomotor, cognitive and reaction time function: Normative data for 18–45 year old

**DOI:** 10.1371/journal.pone.0260351

**Published:** 2021-11-22

**Authors:** Aura Kullmann, Robin C. Ashmore, Alexandr Braverman, Christian Mazur, Hillary Snapp, Erin Williams, Mikhaylo Szczupak, Sara Murphy, Kathryn Marshall, James Crawford, Carey D. Balaban, Michael Hoffer, Alexander Kiderman

**Affiliations:** 1 Neurolign USA LLC, a Subsidiary of Neurolign Technologies Inc. (formerly Neuro Kinetics, Inc.), Pittsburgh, Pennsylvania, United States of America; 2 Department of Statistics and Data Science, The Hebrew University of Jerusalem, Jerusalem, Israel; 3 Department of Otolaryngology, Miller School of Medicine, University of Miami, Miami, Florida, United States of America; 4 Naval Medical Center, San Diego, California, United States of America; 5 Department of Defense, Hearing Center of Excellence, San Antonio, Texas, United States of America; 6 Madigan Army Medical Center, Tacoma, Washington, United States of America; 7 Department of Otolaryngology, University of Pittsburgh, Pittsburgh, Pennsylvania, United States of America; 8 Department of Neurological Surgery, Miller School of Medicine, University of Miami, Miami, Florida, United States of America; Brandenburgische Technische Universitat Cottbus-Senftenberg, GERMANY

## Abstract

Eye movements measured by high precision eye-tracking technology represent a sensitive, objective, and non-invasive method to probe functional neural pathways. Oculomotor tests (e.g., saccades and smooth pursuit), tests that involve cognitive processing (e.g., antisaccade and predictive saccade), and reaction time tests have increasingly been showing utility in the diagnosis and monitoring of mild traumatic brain injury (mTBI) in research settings. Currently, the adoption of these tests into clinical practice is hampered by a lack of a normative data set. The goal of this study was to construct a normative database to be used as a reference for comparing patients’ results. Oculomotor, cognitive, and reaction time tests were administered to male and female volunteers, aged 18–45, who were free of any neurological, vestibular disorders, or other head injuries. Tests were delivered using either a rotatory chair equipped with video-oculography goggles (VOG) or a portable virtual reality-like VOG goggle device with incorporated infrared eye-tracking technology. Statistical analysis revealed no effects of age on test metrics when participant data were divided into pediatric (i.e.,18–21 years, following FDA criteria) and adult (i.e., 21–45 years) groups. Gender (self-reported) had an effect on auditory reaction time, with males being faster than females. Pooled data were used to construct a normative database using 95% reference intervals (RI) with 90% confidence intervals on the upper and lower limits of the RI. The availability of these RIs readily allows clinicians to identify specific metrics that are deficient, therefore aiding in rapid triage, informing and monitoring treatment and/or rehabilitation protocols, and aiding in the return to duty/activity decision. This database is FDA cleared for use in clinical practice (K192186).

## Introduction

Eye movements have been traditionally used as a non-invasive evaluation of neural functions, due to the wide representation of oculomotor nuclei throughout the brain [1, 2]. Recent advances in eye-tracking technology using video-oculographic (VOG) recordings with high resolution cameras allow precise measurement and quantification of linear and torsional eye movements. The four types of eye movements—i.e., saccade, smooth pursuit, vergence, and vestibulo-ocular—can be assessed using a battery of tests that provide visual or motion stimuli to elicit these movements while recording horizontal, vertical, and torsional eye movements. Furthermore, the addition of cognitive (e.g., antisaccade, predictive saccade) and reaction time (e.g., auditory or visual) tasks permits probing of more complex neural processing. For example, antisaccade is a task in which the subject is required to look in the opposite direction of a stimulus appearing after fixation. This is a complex task comprised of several components: subjects must remember the task instructions, inhibit the natural tendency to look towards an appearing target, translate the spatial location of the target to its mirror image location, and execute a saccade to a blank position on the screen. The voluntary inhibition of the reflexive saccade is controlled by nuclei located in the frontal lobe; therefore, this test provides an estimation of injury or dysfunction of the frontal lobe [3].

Numerous studies have shown deficits in eye movements and reaction time in neurodegenerative diseases [4, 5], including Alzheimer’ disease [6], Parkinson’s [7–9] and multiple sclerosis [10–12], psychiatric conditions [13], and more recently following mild traumatic brain injury (mTBI), also known as concussion [14–16]. In the past decade, awareness and understanding of mTBI/concussion has been substantially improved, along with a growing body of research uncovering acute and long-term effects of mild head injuries. It is now understood that it is important to accurately diagnose concussion in a timely manner, manage and provide adequate therapy, and decide when a person is able to return to duty/work/activity [14–16]. While clinicians are aware of this importance, simple tools for diagnosis and monitoring mTBI are still under development and evaluation.

Deficits in eye movements are present in up to 90% of patients with acute mTBI [17–24], thus positioning oculomotor tests as essential tools for evaluating mTBI. Although numerous studies have shown that oculomotor, cognitive, and reaction time tests combined with eye tracking can be beneficial in clinical practice, the adoption of these test in clinical practice is lacking for at least two reasons. First is the availability of a simple tool, preferably portable, with tests that are easy to administer and interpret. There are commercially available, FDA-cleared, portable VOGs goggles equipped with high speed cameras and software that can be used both in clinical settings and on the sidelines of sports, but the technology is used mainly in research settings. Second is lack of standardized normative data, collected from healthy individuals, against which to compare patient results. Some studies do report oculomotor data from healthy individuals [19, 25–27]; however, often the number of subjects tends to be low or the age is not taken into account. In addition, data are reported usually in the format of mean and standard deviation, while in clinical practice, reference intervals are preferred. Establishing a normative database is critical to accurately assess oculomotor, cognitive, and reaction time deficits and functional performance outcomes. Such a database could serve diagnostic purposes and disease monitoring over time, thus aiding not only in diagnosis but also in evaluating treatment efficacy, rehabilitation, and return to duty/work/activity decisions.

The goal of this study was to construct an eye-tracking reference database, defined as data representing the range of performance on a particular test of a group of medically healthy individuals with homogenous demographic distribution [28] for oculomotor, reaction time, and cognitive tests in individuals 18–45 years old, to be used in clinical practice.

## Materials and methods

### Study participants

All research activities were conducted according to the principles expressed in the Declaration of Helsinki, and were approved by the following Institutional Review Boards (IRB): a) University of Miami, IRB# 2015036; b) Naval Medical Center San Diego, IRB# NMCSD.2013.0060; and c) Madigan Army Medical Center, IRB# 393240–1. The trials were registered under NCT02486003 and NCT01832714. The study used written informed consent and only participants who signed the informed consent were enrolled. Participants consisted of 466 adult male and female volunteers, ages 18–45 years old (gender ascertained by self-report; see demographics in Table 1) recruited from three different sites: #1 University of Miami, Miami, FL (n = 166 subjects enrolled in 2015); #2 Naval Medical Center San Diego, San Diego, CA, (n = 50 subjects enrolled between 2013–2015); and #3 Madigan Army Medical Center, Fort Lewis, Washington (n = 250 subjects enrolled between 2013–2015). Participants were recruited from the general population and included non-professional athletes who participate in intercollegiate athletics, civilians, and military service members. This mixture ensures appropriate representation of different levels of activity and skills that are typically encountered in the general population. All three sites used the same inclusion/exclusion criteria listed in S1 Table in the supplemental data. Exclusion criteria included conditions/diseases that could impact the oculomotor, reaction time, and cognitive tests. Specifically, participants with history of brain injury, repeated blast exposure, presence of severe aphasia, history of diagnosed neuropsychiatric disorders (e.g., hypochondriasis, major depression, schizophrenia), neurodegenerative disorders, disorders of hearing and balance (e.g., Meniere’s disease, multiple sclerosis, vestibular neuritis, vestibular schwannoma, sudden sensorineural hearing loss), cerebrovascular disorders, history of ear operation other than myringotomy tube in the past, and systemic disorders (e.g., chronic renal failure, cirrhosis of the liver) were excluded. Special populations including women who were pregnant, children under 18 year old, and those with impaired decision-making capacity were also excluded from study. Data presented here include 300 healthy controls subjects included in earlier papers from this laboratory that described the use of oculomotor, cognitive, and reaction time tests for evaluation of mTBI [17, 21, 29]. Subjects were categorized into two populations based on age ranges defined by the US FDA: pediatric subjects aged 18–21 years and adult subjects aged 22–45 years (Table 1).

**Table 1:** Demographics of the participants.

Age/groups (years)	Mean age (years ±SD)	N	Sex M/F
18–45	24.75 ± 6.27	466	307/159
Group 1: 18–21	19.60 ± 1.07	202	127/75
Group 2: 22–45	28.68 ± 5.73	264	180/84

M = male; F = female; SD = standard deviation, N = number of participants.

### Devices

All eye-tracking technology and software used in this study were developed by Neurolign USA LLC (formerly known as Neuro Kinetics, Inc., Pittsburgh, PA). Two FDA-cleared eye-tracking devices were used in this study: a) Neurolign Dx NOTC, formerly known as I-Portal^®^ Neurotologic Test Center (NOTC) and b) Neurolign Dx 100, formerly known as I-Portal^®^ Portable Assessment System™—Nystagmograph (I-PAS™). The study started using the NOTC initially and then later added the Dx 100 device to the protocol. The devices are substantially equivalent, i.e., both use the same software and have the same high-resolution eye-tracking capabilities. The only differences are in the type of stimuli that each device can deliver and the device physical size. The Neurolign Dx NOTC is a rotation chair that provides rotational, visual, and auditory stimuli to an individual while recording eye movements and reaction time responses. The Neurolign Dx 100 is a portable, compact, 3D, head-mounted display system with integrated eye-tracking that provides visual and auditory stimuli while recording eye movements. Motor reaction times are also recorded via a small, wired, hand-held trigger button box. Both the Neurolign Dx NOTC and the Neurolign Dx 100 use infrared video-oculography (VOG) and the same acquisition and analysis software. High resolution eye-tracking images were acquired via two high-speed digital infrared cameras (940nm; sampling rate 100 frames/sec). Spatial resolution for horizontal and vertical eye-tracking and torsion is <0.1 degrees; eye-tracking range is at least ±30 degrees horizontal, ±20 degrees vertical and ±10 degrees torsional. Data were collected using I-Portal® software which captures, time stamps (critical for synchronization), and analyzes digital images of the eye, collecting horizontal and vertical eye movement data. VEST™ software was used to operate the hardware, manage and capture the stimulus profiles, integrate I-Portal® eye-tracking data, and analyze data, generating a comprehensive set of metrics.

### Battery of tests

Table 2 presents a detailed description of the test battery consisting of nine different tests to assess oculomotor, cognitive, and reaction time metrics. The described test battery has the ability to detect patterns of measurable deficits associated with mTBI and other neurological conditions [17–21, 30, 31], as well as oculomotor, vestibular, and neuro-otological conditions. The complete test battery was performed in 300 participants using the Neurolign Dx NOTC and in 166 participants using the Neurolign Dx 100. All participants were tested only once with the battery of tests listed in Table 2 and the following additional tests not included in this paper, the optokinetic response, subjective visual horizontal and vertical. In addition, participants from the Naval Medical Center San Diego and Madigan Army Medical Center were tested with sinusoidal harmonic acceleration, visual enhancement, visual suppression, and the computerized rotational head impulse test (crHIT) administered in the NOTC device. These data are included in a companion manuscript [32]. The order of the tests was the same for sites # 2 and # 3 (Naval Medical Center San Diego and Madigan Army Medical Center) and slightly different for #1 (University of Miami). In a previous pilot study [33] performed at a different site, we investigated the effects of the examiner and device on test results. Subjects (n = 30, healthy controls) were tested three times with varying time intervals between each session, ranging from 0.2h to 48h, using 3 different Dx 100 (I-PAS) devices and 5 different examiners. The order of the tests was different in each session. The results indicated that subjects’ responses are not affected by either the examiner, the device or test order [33].

**Table 2:** Battery of oculomotor, cognitive, and reaction time tests.

	Tests	Metrics
1	Saccade—Random, Horizontal (SH): subject follows a dot displayed 15 times at pseudo-randomly distributed times (between 1 to 2 seconds) and pseudo-random displacements on a horizontal plane (-30 to +30 degrees).	a) Latency (s) = time from stimulus presentation until saccade is initiated. Data are presented as an average of all saccade onset latencies.
		b) Accuracy (%) = difference between eye position and stimulus position for the main saccade, expressed in percentage relative to stimulus position. Data are presented as an average of all main saccade accuracies.
		c) Final Accuracy (%) = difference between eye position and stimulus position for the final position, including corrective saccades, expressed in percentage relative to stimulus position. Data are presented as an average of all saccade accuracies.
		d) Area Under Main Sequence Fit (AUF) (deg^2^/s). Eye velocity is plotted as a function of saccade displacement and fitted with an exponential function. To evaluate the overall velocity and amplitude relationship, the software computes the area under the curve, out to 30 degrees of eye displacement = AUF.
		e) Peak velocity = eye velocity corresponding to each eye displacement in response to a stimulus displacement
2	Saccade—Random, Vertical (SV): subject follows a dot displayed 15 times at pseudo-randomly distributed times (between 1 to 2 seconds) and pseudo-random displacements on a vertical plane (-20 to +20 degrees).	same metrics as above
3	Smooth Pursuit: subject follows a dot as it displaced (moves) sinusoidally horizontally then vertically at different speeds:	a) Velocity Gain = ratio between the slow phase component of eye velocity and pursuit tracker stimuli. Data are averaged for the leftward and rightward moving stimuli.
	Smooth Pursuit—Horizontal 0.1 Hz, 2 cycles	
		b) Asymmetry = Velocity Gain Asymmetry; represents the difference between gain calculated for leftward and rightward moving stimuli
		c) Position Gain = ratio between the slow phase component of eye velocity and pursuit tracker stimuli
		d) Saccadic component (%) = percentage of eye movement spent on a saccadic movement versus pursuit movement
	Smooth Pursuit—Horizontal 0.75Hz, 6 cycles	Same as above
4	Smooth Pursuit—Vertical 0.1Hz, 2 cycles	Same as test #3
	Smooth Pursuit—Vertical 0.75Hz, 6 cycles	Same as test #3
5	Predictive Saccades: Subject is directed to follow a dot as it is displayed. Subject is presented with 6 pseudo-random saccade stimuli followed by 20 mirrored saccade stimuli with a repeated displacement +/-10 degree, horizontal, at a constant time interval of 0.65 seconds.	Percentage predicted (%) = percentage of predicted saccades
6	Antisaccades: Subject is required to fixate on a central target for 1.5 to 2.5 seconds, after which a peripheral target is presented. Subject is required to generate an eye movement of the same distance as the target displacement, but in the exact opposite direction. There are 20 anti-saccades with time between saccades randomly selected from 1 to 2 seconds and random displacement between -24 to + 24 degrees.	Error Rate (%) = percentage of prosaccade errors, i.e. where the subject looks toward rather than away from the stimulus
7	Visual Reaction Time: 20 light stimuli are presented in the center of the screen, with a random timing. The subject is directed to signal their recognition by pressing a button.	Latency (ms) = time difference from stimulus presentation until button is pressed
8	Auditory Reaction Time: 20 sound stimuli are presented with a random timing. The subject is directed to signal their recognition by pressing a button.	Latency (ms) = time difference from stimulus presentation until button is pressed
9	Saccade and Reaction Time: 30 visual saccadic stimuli are randomly projected every 1 to 2 seconds with a displacement of -24 to + 24 degrees. The participants are directed to gaze at the saccadic stimulus and then press either the left or right button to record whether the stimulus was projected to the right or to the left.	Saccadic metrics–are same as in test #1.
		a) Latency (s)
		b) Accuracy (%)
		c) Final Accuracy (%)
		Motor reaction time metrics:
		d) Latency mean (s)–for Left Button = time difference from stimulus presentation until the left button is pressed
		e) Latency mean (s)–for Right Button = time difference from stimulus presentation until the right button is pressed

Description of each test and metrics measured for that test.

### Data analysis

Acquired data for each test were inspected for completion and validity and analyzed in VEST™ software, with results exported for statistical analysis (see below).

#### Treatment of artifacts and outlying samples

Data were filtered, or partially removed, on a test-by-test basis by manual adjustment of VEST™ software controls according to standard operating guidelines/procedures for the removal of artifacts (e.g., blinks, recording noise, temporary failures of eye-tracking, shifting of goggles, erroneous responses or those unrelated to the task) to separate eye movement signals from other recording noise, or to segregate saccadic activity from pursuit activity. Individual tests for some participants were removed from analysis when the data quality was judged to be inadequate for accurate measurement or produced analytic errors, or test was not run.

#### Statistical analysis

Within each test, a number of metrics describing specific components of eye movement were calculated by VEST™ software and exported to IBM SPSS Statistics for Windows, version 21 (IBM Corp., Armonk, N.Y., USA) and Microsoft Excel for further analysis. The statistic results were reported based on VEST measurements.

#### Age and gender effect

To construct a normative database for the test variables (e.g., computing the reference intervals), we first examined the homogeneity of the participants’ test results. In particular, we examined whether the test results are affected by the age and/or gender of the participants. For this purpose, the data was grouped by (a) *age* into two populations: pediatric subjects aged 18–21 years and adult subjects aged 22–45 years (age ranges defined by the US FDA), and (b) *gender* (ascertained by self-report): female and male. The effect of age and gender was tested using the t-test (mean difference) for independent samples and by applying one and two-way (with interaction) analysis of variance (ANOVA). The t-test and one-way ANOVA were used to examine each effect separately, whereas a two-way ANOVA was used to test jointly the effect of age and gender when the interaction between the two was considered as well (i.e., age group×gender). Levene’s test for equal variance was used to decide whether to apply t-test that assumes equal variances or not.

#### Calculation of 95% reference interval (RI) with 90% confidence interval (CI)

For each variable, a 95% RI with a 90% CI on the lower and upper limits of the RI was established assuming a non-parametric distribution, according to the FDA suggested guidelines and other published references [34, 35]. In order to construct the RI, individual metrics were organized by rank from the lowest to the highest value: y_1_, y_2_, …y_N_, where *N* is sample size. The 100(1-α)% RI is given by the upper and lower limits, R_*L*_ and R_*U*_, calculated as follows: RL=y[N×(α2)] and RU=y[N×(1−α2)] with *α* = 0.05 (square brackets indicate the number is rounded to the nearest integer). For the 90% CI for the upper or lower limits of the RI, the 100(1-γ)% limits for CI, (γ = 0.1 for 90% CI), named R_q_ and S_q_, for each R_L_ and R_U_, were calculated [34, 35].

The percentiles 2.5, 5, 10, 25, 75, 90, 95 and 97.5 for each test metric were calculated using a SPSS built in function.

#### One sided metrics

There are a number of metrics for which the lower or the upper limits are either meaningless or are not of clinical interest for the general population. For example, for saccade latency (i.e., the time it takes for the eye to reach the target), the lower limit, 2.5^th^ percentile, is not of clinical interest for the general population, because unusually fast saccades are likely not indicative of any clinical condition. The upper limit, however, is of interest because values greater than this number imply that the eye velocity to reach the target is very slow, which may have clinical implications. For these metrics, the 5^th^ or 95^th^ percentile was calculated for the limit of interest and the limit of no interest was marked with not applicable (n/a) [34, 35].

#### Methods for calculating ‘Peak Velocity’ in the saccade test

During a saccade, eye velocity corresponding to each eye displacement in response to a stimulus displacement was computed by VEST™ software and data were imported in Excel. Data from each subject were placed in bins of 5 deg based on eye displacement; i.e. saccades for which eye displacement was between 30 and 25 deg were grouped together, with the same for 25–20 deg, 20–15 deg, etc. Data were then treated as described above by ranking the values from smallest to the largest and calculating RI and CI as described above.

## Results

Data presented here are from 466 participants, 307 males and 159 females with a mean age of 24.75 ± 6.27 (range 18–45 years) (see Table 1 for demographics). Previous research has shown that most oculomotor, cognitive, and reaction time metrics mature before age 18, are stable between 18–45, and decline thereafter [3, 36–42]. Therefore, this database was limited to this age range. The database includes 9 oculomotor, cognitive, and reaction time tests: horizontal and vertical saccades, horizontal and vertical smooth pursuit, predictive saccades, antisaccades, auditory reaction time, visual reaction time, and saccade and reaction time (Table 2). From each test, a number of metrics describing different components of eye movement (e.g., amplitude, velocity, gain) were computed. The description of each metric is included in Table 2.

### Age and gender effects

The US FDA considers the pediatric population to be 0 to 21-years-old, and the Journal of Academy of Pediatrics terms them as "Late Adolescents” [43]. Thus, pediatric participants (ages 18–21 years) and adult participants (ages 22–45 years) were analyzed to determine whether age has any effect on test results (Table 3). Gender (self-reported) was also examined (Table 3). The findings indicate that with the exception of a few oculomotor, cognitive, and reaction time test metrics, the effect of age or gender was not statistically significant at the 0.05 level. However, with the exception of one test (namely the auditory reaction time test), the statistical effect does not suggest clinical significance, neither for age, nor for gender, because for each metric, the 95% RI with the 90% CI for each age group or gender group showed great overlap. For example, in the test ‘Saccade–Random Vertical’, for the metric ‘latency’, the 95% RIs with the 90% CIs were (sec): 0.22 (0.22–0.23) and 0.22 (0.22–0.23), for the 18-21- and 21–45-year-old groups, respectively. Based on this analysis, data from all participants were pooled for calculation of the RI limits and percentiles, presented in Tables 4–6 and supplemental data. Tables 4–6 present the 95% RI with a 90% CI on the lower and upper limits of the RI for all tests and metrics. Supplemental data S2–S4 Tables present the 2.5, 5, 10, 25, 75, 90, 95 and 97.5 percentile for each metric within each test, and S5 Table presents mean and standard deviation.

**Table 3:** Testing the effect of age group, gender, and interaction between the two on the test metrics using one and two-way analysis of variance (ANOVA).

Test	Variables	(1)		(2)		(3)					
		Age group		Gender		Age group		Gender		Age×Gender	
		F-stat	*p*-value	F-stat	*p*-value	F-stat	*p*-value	F-stat	*p*-value	F-stat	*p*-value
Saccade–Random, Horizontal	Latency (s)	0.512	0.475	2.198	0.139	1.699	0.193	1.903	0.168	2.763	0.097
	Accuracy (%)	1.691	0.194	1.485	0.224	1.299	0.255	1.273	0.260	0.007	0.933
	Final accuracy (%)	1.211	0.272	0.160	0.689	0.305	0.581	0.025	0.875	2.287	0.131
	AUF (deg^2^/s)	1.626	0.203	0.020	0.888	1.322	0.251	0.002	0.967	0.033	0.856
	Peak velocity (25–30°/s)	0.001	0.970	2.871	0.093	0.001	0.982	2.318	0.131	0.028	0.868
Saccade–Random, Vertical	Latency (s)	8.416	0.004	0.000	0.986	12.430	0.000	0.003	0.958	5.773	0.017
	Accuracy (%)	0.037	0.847	0.905	0.342	0.048	0.827	0.940	0.333	0.008	0.928
	Final accuracy (%)	0.356	0.551	0.299	0.585	0.376	0.540	0.511	0.475	0.029	0.866
	AUF (deg^2^/s)	12.198	0.001	1.884	0.171	11.755	0.001	1.456	0.228	0.381	0.538
	Peak velocity (25-30deg/s)	0.002	0.967	0.491	0.485	0.070	0.792	0.000	0.993	1.112	0.294
Smooth Pursuit–Horizontal 0.1Hz	Velocity gain	0.078	0.779	0.888	0.346	0.064	0.801	0.962	0.327	0.062	0.804
	Asymmetry (%)	2.059	0.152	0.484	0.487	2.007	0.157	0.603	0.438	0.001	0.974
	Position gain	4.351	0.038	0.518	0.472	3.495	0.062	0.882	0.348	0.350	0.555
	Saccadic component (%)	0.010	0.921	2.055	0.152	0.058	0.811	2.509	0.114	2.070	0.151
Smooth Pursuit–Horizontal 0.75 Hz	Velocity gain	0.016	0.898	5.980	0.015	0.041	0.840	6.337	0.012	0.695	0.405
	Asymmetry (%)	0.143	0.706	0.710	0.400	0.391	0.532	0.830	0.363	1.113	0.292
	Position gain	13.658	0.000	2.349	0.126	14.923	0.000	2.729	0.099	0.671	0.413
	Saccadic component (%)	2.878	0.090	0.847	0.358	1.414	0.235	0.464	0.496	1.312	0.253
Smooth Pursuit–Vertical 0.1 Hz	Velocity gain	2.122	0.146	0.009	0.924	1.506	0.220	0.035	0.853	0.003	0.958
	Asymmetry (%)	1.871	0.172	1.567	0.211	1.757	0.186	2.164	0.142	0.061	0.805
	Position gain	16.354	0.000	0.086	0.770	14.096	0.000	0.270	0.604	0.057	0.811
	Saccadic component (%)	4.032	0.045	3.654	0.057	3.250	0.072	3.133	0.077	0.002	0.964
Smooth Pursuit–Vertical 0.75 Hz	Velocity gain	0.500	0.480	3.727	0.054	0.179	0.672	3.313	0.069	0.218	0.641
	Asymmetry (%)	6.709	0.010	2.177	0.141	3.775	0.053	1.411	0.236	1.727	0.190
	Position gain	4.317	0.038	0.000	0.983	6.335	0.012	0.000	0.990	2.995	0.084
	Saccadic component (%)	5.479	0.020	11.492	0.001	3.486	0.063	10.010	0.002	0.349	0.555
Predictive Saccades	Percentage predicted (%)	35.909	0.000	0.043	0.835	28.757	0.000	0.057	0.812	1.515	0.219
Antisaccades	Error rate (%)	4.780	0.029	2.799	0.095	0.060	0.806	2.281	0.132	3.649	0.057
Visual Reaction Time	Latency (ms)	0.834	0.362	12.277	0.001	2.041	0.154	0.080	0.778	3.168	0.076
Auditory Reaction Time	Latency (ms)	0.018	0.894	29.000	0.000	0.815	0.367	27.972	0.000	0.593	0.442
Saccade and Reaction Time–Saccade metrics:	Latency (ms)	0.547	0.460	0.406	0.524	0.550	0.459	0.370	0.543	0.067	0.795
	Accuracy (%)	0.822	0.365	1.623	0.203	0.371	0.543	1.317	0.252	0.348	0.556
	Final accuracy (%)	0.008	0.927	1.696	0.193	0.007	0.935	1.765	0.185	0.071	0.790
Saccade and Reaction Time–Motor response:	Latency mean (s), Left	0.020	0.887	0.190	0.664	0.019	0.892	0.103	0.749	0.004	0.949
	Latency mean (s), Right	0.007	0.932	0.060	0.807	0.000	0.995	0.087	0.768	0.030	0.864

The t-test-based results examining the effect of age and gender are not reported here. However, these results are consistent with those of one-way ANOVA.

**Table 4:** Normative data for oculomotor tests: Saccades and smooth pursuit tests.

Test	Metric	RI lower limit	RI upper limit	90% CI for lower limit RI	90% CI for upper limit RI
Saccade–Random, Horizontal	Latency (s)	n/a	0.22	n/a	0.21–0.22
	Accuracy (%)	81	103	80–82	101–105
	Final accuracy (%)	89	104	87–89	102–106
	AUF (deg^2^/s)	8239	n/a	7706–8351	n/a
	Peak Velocity (deg/s) for eye displacement of:				
	30 (deg)	356	n/a	355–374	n/a
	25 (deg)	322	n/a	314–332	n/a
	20 (deg)	301	n/a	292–303	n/a
	15 (deg)	272	n/a	265–278	n/a
	10 (deg)	241	n/a	235–242	n/a
	5 (deg)	121	n/a	117–124	n/a
Saccade–Random, Vertical	Latency (s)	n/a	0.23	n/a	0.22–0.23
	Accuracy (%)	75	109	73–76	108–112
	Final accuracy (%)	79	107	78–80	106–110
	AUF (deg^2^/s)	7630	n/a	6937–7855	n/a
	Peak Velocity (deg/s) for eye displacement of:				
	30 (deg)	337	n/a	323–343	n/a
	25 (deg)	287		280–294	
	20 (deg)	272		268–278	
	15 (deg)	238		235–245	
	10 (deg)	191		184–198	
	5 (deg)	101		100–104	
Smooth Pursuit–Horizontal 0.1Hz	Velocity gain	0.78	1.07	0.76–0.80	1.07–1.08
	Asymmetry (%)	-8.80	7.53	(-9.91)–(-8.17)	7.26–7.91
	Position gain	0.96	1.04	0.95–0.96	1.04–1.04
	Saccadic component (%)	n/a	35	n/a	34–37
Smooth Pursuit—Horizontal 0.75 Hz	Velocity gain	0.62	1.08	0.58–0.71	1.08–1.09
	Asymmetry (%)	-8.93	9.00	(-14.52)–(-5.74)	8.05–9.93
	Position gain	0.79	1.10	0.77–0.82	1.09–1.12
	Saccadic component (%)	n/a	37	n/a	34–40
Smooth Pursuit—Vertical 0.1 Hz	Velocity gain	0.69	1.07	0.64–0.71	1.06–1.09
	Asymmetry (%)	-12.36	11.46	(-13.79)–(-11.62)	9.91–13.50
	Position gain	0.95	1.07	0.94–0.95	1.06–1.07
	% saccadic component	n/a	32	n/a	29–34
Smooth Pursuit—Vertical 0.75 Hz	Velocity gain	0.42	1.09	0.37–0.43	1.07–1.10
	Asymmetry (%)	-23.43	29.01	(-26.75)–(-21.57)	27.78–34.11
	Position gain	0.73	1.11	0.73–0.75	1.10–1.18
	% saccadic component	n/a	52	n/a	50–53

The upper and lower limits of the reference interval (RI) and 90% confidence interval (CI) for each limit are presented. For one-sided metrics, the limit of no interest is marked with not applicable (n/a). For description of each metric see Table 2.

**Table 5:** Normative data for tests with cognitive involvement: Predictive saccades and antisaccades.

Test	Metric	RI lower limit	RI upper limit	90% CI for lower limit RI	90% CI for upper limit RI
Predictive Saccades	Percentage predicted (%)	17	n/a	14–17	n/a
Antisaccades	Error Rate (%) = % of pro-saccade errors	0	50	0–0	50.00–50.00

The upper and lower limits of the reference interval (RI) and 90% confidence interval (CI) for each limit are presented. For one-sided metrics, the limit of no interest is marked with not applicable (n/a). For description of each metric see Table 2.

**Table 6:** Normative data for reaction time tests.

Test	Metric	RI lower limit	RI upper limit	90% CI for lower limit RI	90% CI for upper limit RI
Visual Reaction Time	Latency (ms)	n/a	333.55	n/a	323.330–338.33
Auditory Reaction Time	Latency (ms)	n/a	316.37	n/a	305.56–330.00
	Female—Latency (ms)	n/a	335.56	n/a	320.00–346.67
	Male—Latency (ms)	n/a	305.00	n/a	296.43–316.67
Saccade and Reaction Time	**Saccade metrics:**				
	Latency (ms)	0.14	0.28	0.14–0.15	0.28–0.30
	Accuracy (%)	74	101	65–75	100–105
	Final accuracy (%)	79	106	72–82	103–112
	**Motor response metrics:**				
	Latency mean (s)–for Left Button	n/a	0.63	n/a	0.58–0.65
	Latency mean (s)–for Right Button	n/a	0.58	n/a	0.56–0.65

The upper and lower limits of the reference interval (RI) and 90% confidence interval (CI) for each limit are presented. For one-sided metrics, the limit of no interest is marked with not applicable (n/a). For description of each metric see Table 2.

Analysis by self-reported gender indicated that gender had an influence on auditory reaction time, with males being significantly faster than females (Table 6).

## Discussion

Tracking of eye movements is a well-established, non-invasive method for assessing many neurocognitive functions, and has been shown to be sensitive to detecting changes associated with traumatic brain injury, neurodegenerative diseases, and other dysfunctions [4, 14]. Previous studies from our laboratories and others have demonstrated a role for oculomotor, cognitive, and reaction time tests in the detection, diagnosis, and monitoring of mTBI [17, 20, 21, 24, 44–46]. The utility of these tests has been also shown in identification of deficits Alzheimer’s disease [6], Parkinson’s [7–9], multiple sclerosis [10–12] and other conditions.

Several studies have published data form healthy persons, albeit the sample size is relatively modest, age intervals are different, and most results are presented as mean and standard deviation, rather than reference intervals. Saccade latency (for horizontal saccades) varied among studies, with values including 180 ± 30 ms (age 20.6 ± 1.8) in Cochrane et al. 2019 [19], 237.24 ± 18.23 ms (age 18–30) in Seferlis et al. 2015 [27], 180 ± 30 ms (age 6–76; rightward 30 degree saccades) in Hopf et al. 2018 [26], and to 170 ± 20 ms (age 18–45) in the present study (S5 Table included in the supplemental data). Saccade accuracy (for horizontal saccades) was 97.1 ± 5.5% in Cochrane et al. 2019 [19], 91.40 ± 14.27% in Seferlis et al. 2015 [27], and 87 ± 6% in Hopf et al. 2018 [26], and 92.11 ± 5.12% in the present study (S5 Table). Smooth pursuit gain values, for horizontal and vertical smooth pursuit at 0.1 Hz, were 0.97 ± 0.07% and 0.93 ± 0.12%, respectively in Cochrane et al. 2019 [19], and 0.95 ± 0.10% and 0.90 ± 0.10%, respectively in the present study (S5 Table). Using similar equipment as the one used in this study, the auditory and visual reaction time values were 246 ms (ranges 143–552 ms) and 273 ms (ranges 169–507 ms), respectively in Cochrane et al. 2019 [19], and 224.32 ± 39.71 ms and 270.17 ± 30.13 in our study (S5 Table). While most values are similar, the differences may be attributed to age or methodology.

These studies along with others [25, 37, 47], indicate the need for normative data for oculomotor, cognitive, and reaction time tests. The present study, by providing an FDA-cleared normative database, which reports the data in the format of reference intervals, may facilitate the adoption of these tests in clinical practice.

### Neuro-anatomical pathways employed by oculomotor, cognitive, and reaction time tests

Oculomotor, cognitive, and reaction time tests probe the function of multiple neural pathways involved in the control of eye movement, vestibular function, reaction time, and motor and cognitive processing [2]. For example, saccades engage the frontal eye field area [1, 2], reaction time tests engage networks in the superior temporal cortex, premotor cortex, and cerebellum [48], whereas antisaccades or predictive saccades engage additional executive function and attention networks [3, 49]. Moreover, within each test, different components of planning and execution of eye or hand movements may engage different pathways. As such, saccade latency, which is the duration that it takes for the eye to initiate a movement towards the target, is controlled by central and cortical structures, while speed of eye movement and accuracy, i.e. how fast and how well the eyes land on the target, are controlled by both cortical and premotor structures at the brainstem level where the motor command is initiated [2]. The speed and amplitude of the saccades may involve the saccade burst generator located in the paramedian pontine reticular formation in the brainstem, which receives direct and/or indirect input from cortical structures (e.g., frontal eye field, parietal cortex), superior colliculus, and cerebellum [50, 51]. Thus, our results, by providing detailed normative data for different components of eye (and/or hand) movements, may constitute the basis for understanding and evaluating disturbances of many neural pathways as a result of various neurological conditions.

### Role of oculomotor, cognitive, and reaction time tests and normative database in mTBI

mTBI, also known as concussion, results in injuries to brain pathways that control or influence oculomotor behaviors, reaction time responses, and cognitive processes. In the last decade, a plethora of research has been dedicated to identifying oculomotor deficits in mTBI, and using oculomotor testing as an aid in diagnosis of acute mTBI [14]. These studies suggest that up to 90% of patients with acute mTBI present with deficits in one or more oculomotor, cognitive, and reaction time tests, such as deficits in saccades, smooth pursuit, reaction time, and tasks involving cognitive processing [17, 19, 21, 24, 52, 53]. In addition, certain oculomotor deficits, e.g., saccades, reaction time, persist long after the initial injury [20, 24, 44]. Our previous work in mTBI indicated that a set of 6–8 metrics extracted from oculomotor tests, e.g., saccades, antisaccades, smooth pursuit, and vestibular tests, was strongly associated with the presence of a concussion in both acute and chronic patients [17, 22]. These tests were also able to monitor progression over time after mTBI [21] and response to treatment [54]. For example, impairments in saccades (i.e., saccadic intrusions) following mTBI can cause visual disturbances (e.g., double vision, oscillopsia) accompanied by headaches. Improvements in saccadic eye movements using vision therapy (and/or medication) mitigated clinical symptoms [54].

This extensive research clearly highlights the usefulness of oculomotor testing in mTBI and other neurological condition. Thus, the availability of an oculomotor, cognitive, and reaction time normative database that is FDA-cleared, will have a significant impact in the adoption of oculomotor testing in clinical practice. This normative database eliminates the need for individual baseline pre-injury data, which is often unavailable, and instead provides the normative ranges for healthy individuals as a reference with which to compare patient data. In particular for mTBI, this database, as well as the normative database for vestibular tests [32], may aid clinicians in diagnosing, monitoring the recovery, making decisions regarding patient return to duty/work/activity, and assessing the effects of pharmacological treatments and/or rehabilitation protocols.

## Conclusions

Recent advances in clinical eye-tracking technology, its non-invasive nature, the development of software and technology that provide precise and objective measures, and the batteries of tests that target not only the oculomotor systems, but importantly other motor, vestibular and cognitive functions (e.g., attention, cognition, decision making), propelled these methods as essential tools (biomarkers) for evaluating mTBI/concussion. The availability of an FDA-cleared oculomotor, cognitive, and reaction time normative database aids clinicians in diagnosis, monitoring of treatment and rehabilitation protocols, and decisions concerning return to duty/work/activity. In addition, a normative database of this form has widespread applications not only for mTBI/concussion but also other neurological conditions, e.g., neurodegenerative diseases. The current limitation of this database is the age range, 18–45. Future studies are necessary to expand the database to other age ranges.

## Supporting information

S1 Table Inclusion/exclusion criteria. (PDF)

S2 Table Percentiles for saccades and smooth pursuit tests. Data present the 2.5, 5, 10, 25, 75, 90, 95 and 97.5 percentile for each metric.
(PDF)

S3 Table Percentiles for predictive saccades and antisaccades. Data represent the 2.5, 5, 10, 25, 75, 90, 95 and 97.5 percentile for each metric.
(PDF)

S4 Table Percentiles for reaction time tests. Data represent the 2.5, 5, 10, 25, 75, 90, 95 and 97.5 percentile for each metric.
(PDF)

S5 Table Descriptive statistics. Mean and standard deviation (SD) is presented for each test metric.
(PDF)

## References

[1] Vernet M, Quentin R, Chanes L, Mitsumasu A, Valero-Cabre A. Frontal eye field, where art thou? Anatomy, function, and non-invasive manipulation of frontal regions involved in eye movements and associated cognitive operations. Front Integr Neurosci. 2014;8:66. Epub 2014/09/10. doi: 10.3389/fnint.2014.00066 ; PubMed Central PMCID: PMC4141567. 25202241 PMC4141567

[2] Leigh RJ, Zee DS. The Neurology of Eye Movement. Oxford University Press, New York, NY, USA, 2006.

[3] Crawford TJ, Smith ES, Berry DM. Eye Gaze and Aging: Selective and Combined Effects of Working Memory and Inhibitory Control. Front Hum Neurosci. 2017;11:563. Epub 2017/12/13. doi: 10.3389/fnhum.2017.00563 ; PubMed Central PMCID: PMC5711774. 29230169 PMC5711774

[4] MacAskill MR, Anderson TJ. Eye movements in neurodegenerative diseases. Curr Opin Neurol. 2016;29(1):61–8. doi: 10.1097/WCO.0000000000000274 . 26641817

[5] Serra A, Chisari CG, Matta M. Eye Movement Abnormalities in Multiple Sclerosis: Pathogenesis, Modeling, and Treatment. Front Neurol. 2018;9:31. Epub 2018/02/23. doi: 10.3389/fneur.2018.00031 ; PubMed Central PMCID: PMC5807658. 29467711 PMC5807658

[6] Molitor RJ, Ko PC, Ally BA. Eye movements in Alzheimer’s disease. J Alzheimers Dis. 2015;44(1):1–12. doi: 10.3233/JAD-141173 ; PubMed Central PMCID: PMC5332166. 25182738 PMC5332166

[7] Pretegiani E, Optican LM. Eye Movements in Parkinson’s Disease and Inherited Parkinsonian Syndromes. Front Neurol. 2017;8:592. doi: 10.3389/fneur.2017.00592 ; PubMed Central PMCID: PMC5684125. 29170650 PMC5684125

[8] Vintonyak O, Gorges M, Muller HP, Pinkhardt EH, Ludolph AC, Huppertz HJ, et al. Patterns of Eye Movement Impairment Correlate with Regional Brain Atrophy in Neurodegenerative Parkinsonism. Neurodegener Dis. 2017;17(4–5):117–26. doi: 10.1159/000454880 . 28268209

[9] Wu CC, Cao B, Dali V, Gagliardi C, Barthelemy OJ, Salazar RD, et al. Eye movement control during visual pursuit in Parkinson’s disease. PeerJ. 2018;6:e5442. doi: 10.7717/peerj.5442 ; PubMed Central PMCID: PMC6109371. 30155357 PMC6109371

[10] Alpini D, Milanese C, Berardi C. Value of otoneurological tests in the staging of multiple sclerosis. Ital J Neurol Sci. 1987;Suppl 6:103–8. Epub 1987/06/01. . 3654169

[11] De Santi L, Lanzafame P, Spano B, D’Aleo G, Bramanti A, Bramanti P, et al. Pursuit ocular movements in multiple sclerosis: a video-based eye-tracking study. Neurol Sci. 2011;32(1):67–71. Epub 2010/08/24. doi: 10.1007/s10072-010-0395-1 . 20730462

[12] Nij Bijvank JA, Petzold A, Coric D, Tan HS, Uitdehaag BMJ, Balk LJ, et al. Saccadic delay in multiple sclerosis: A quantitative description. Vision Res. 2020;168:33–41. Epub 2020/02/18. doi: 10.1016/j.visres.2020.01.003 . 32065930

[13] O’Driscoll GA, Callahan BL. Smooth pursuit in schizophrenia: a meta-analytic review of research since 1993. Brain Cogn. 2008;68(3):359–70. Epub 2008/10/11. doi: 10.1016/j.bandc.2008.08.023 . 18845372

[14] Stuart S, Parrington L, Martini D, Peterka R, J C, L K. The Measurement of Eye Movements in Mild Traumatic Brain Injury: A Structured Review of an Emerging Area. Front Sports Act Living 2020;2:5. doi: 10.3389/fspor.2020.00005 33345000 PMC7739790

[15] Feller CN, Goldenberg M, Asselin PD, Merchant-Borna K, Abar B, Jones CMC, et al. Classification of Comprehensive Neuro-Ophthalmologic Measures of Postacute Concussion. JAMA Netw Open. 2021;4(3):e210599. doi: 10.1001/jamanetworkopen.2021.0599 ; PubMed Central PMCID: PMC7930925. 33656530 PMC7930925

[16] Schepart Z, Putukian M. Sideline assessment of concussion. Handb Clin Neurol. 2018;158:75–80. doi: 10.1016/B978-0-444-63954-7.00008-2 . 30482377

[17] Balaban C, Hoffer ME, Szczupak M, Snapp H, Crawford J, Murphy S, et al. Oculomotor, Vestibular, and Reaction Time Tests in Mild Traumatic Brain Injury. PLoS One. 2016;11(9):e0162168. Epub 2016/09/23. doi: 10.1371/journal.pone.0162168 ; PubMed Central PMCID: PMC5031310 27654131 PMC5031310

[18] Ciuffreda KJ, Kapoor N, Rutner D, Suchoff IB, Han ME, Craig S. Occurrence of oculomotor dysfunctions in acquired brain injury: a retrospective analysis. Optometry. 2007;78(4):155–61. Epub 2007/04/03. doi: 10.1016/j.optm.2006.11.011 . 17400136

[19] Cochrane GD, Christy JB, Almutairi A, Busettini C, Swanson MW, Weise KK. Visuo-oculomotor Function and Reaction Times in Athletes with and without Concussion. Optometry and vision science: official publication of the American Academy of Optometry. 2019;96(4):256–65. Epub 2019/03/26. doi: 10.1097/opx.0000000000001364 ; PubMed Central PMCID: PMC6445703. 30907863 PMC6445703

[20] Danna-Dos-Santos A, Mohapatra S, Santos M, Degani AM. Long-term effects of mild traumatic brain injuries to oculomotor tracking performances and reaction times to simple environmental stimuli. Sci Rep. 2018;8(1):4583. Epub 2018/03/17. doi: 10.1038/s41598-018-22825-5 ; PubMed Central PMCID: PMC5854576. 29545567 PMC5854576

[21] Hoffer ME, Balaban C, Szczupak M, Buskirk J, Snapp H, Crawford J, et al. The use of oculomotor, vestibular, and reaction time tests to assess mild traumatic brain injury (mTBI) over time. Laryngoscope Investig Otolaryngol. 2017;2(4):157–65. Epub 2017/09/13. doi: 10.1002/lio2.74 ; PubMed Central PMCID: PMC5562938. 28894835 PMC5562938

[22] Kelly KM, Kiderman A, Akhavan S, Quigley MR, Snell ED, Happ E, et al. Oculomotor, Vestibular, and Reaction Time Effects of Sports-Related Concussion: Video-Oculography in Assessing Sports-Related Concussion. The Journal of head trauma rehabilitation. 2019;34(3):176–88. Epub 2018/09/21. doi: 10.1097/HTR.0000000000000437 ; PubMed Central PMCID: PMC6553977. 30234848 PMC6553977

[23] Stuss DT, Stethem LL, Hugenholtz H, Picton T, Pivik J, Richard MT. Reaction time after head injury: fatigue, divided and focused attention, and consistency of performance. J Neurol Neurosurg Psychiatry. 1989;52(6):742–8. Epub 1989/06/01. doi: 10.1136/jnnp.52.6.742 ; PubMed Central PMCID: PMC1032026. 2746267 PMC1032026

[24] King JE, Pape MM, Kodosky PN. Vestibular Test Patterns in the NICoE Intensive Outpatient Program Patient Population. Mil Med. 2018;183(suppl_1):237–44. Epub 2018/04/11. doi: 10.1093/milmed/usx170 . 29635576

[25] Bucsuházy K, Semela M. Case Study: Reaction Time of Children According to Age. Procedia Engineering 2017;187 (2017):408–13. doi: 10.1016/j.proeng.2017.04.393

[26] Hopf S, Liesenfeld M, Schmidtmann I, Ashayer S, Pitz S. Age dependent normative data of vertical and horizontal reflexive saccades. PLoS One. 2018;13(9):e0204008. doi: 10.1371/journal.pone.0204008 ; PubMed Central PMCID: PMC6143243. 30226877 PMC6143243

[27] Seferlis F, Chimona TS, Papadakis CE, Bizakis J, Triaridis S, Skoulakis C. Age related changes in ocular motor testing in healthy subjects. J Vestib Res. 2015;25(2):57–66. doi: 10.3233/VES-150548 . 26410670

[28] Kendall PC, Sheldrick RC. Normative data for normative comparisons. J Consult Clin Psychol. 2000;68(5):767–73. Epub 2000/11/09. . 11068962

[29] Hoffer ME, Levin BE, Snapp H, Buskirk J, Balaban C. Acute findings in an acquired neurosensory dysfunction. Laryngoscope Investig Otolaryngol. 2019;4(1):124–31. doi: 10.1002/lio2.231 ; PubMed Central PMCID: PMC6383299. 30828629 PMC6383299

[30] Bin Zahid A, Hubbard ME, Lockyer J, Podolak O, Dammavalam VM, Grady M, et al. Eye Tracking as a Biomarker for Concussion in Children. Clin J Sport Med. 2018. Epub 2018/08/11. doi: 10.1097/JSM.0000000000000639 . 30095503

[31] Heitger MH, Anderson TJ, Jones RD, Dalrymple-Alford JC, Frampton CM, Ardagh MW. Eye movement and visuomotor arm movement deficits following mild closed head injury. Brain. 2004;127(Pt 3):575–90. Epub 2004/01/23. doi: 10.1093/brain/awh066 . 14736751

[32] Kullmann A, Ashmore RC, Braverman A, Mazur C, Snapp H, Williams E, et al. Normative data for ages 18–45 for ocular motor and vestibular testing using eye tracking. Laryngoscope Investig Otolaryngol. 2021;6(5):1116–27. doi: 10.1002/lio2.632 ; PubMed Central PMCID: PMC8513422. 34667856 PMC8513422

[33] Kiderman A, Gonzalez J, Gallagher CW, Whinna AM. Test-retest Repeatability and Reproducibility of Oculomotor, Vestibular, and Reaction Time Testing in 3D Head Mounted Display (HMD) with Integrated Eye Tracking. National Neurotrauma Societ; Santa Fe, New Mexico2015.

[34] Horn PS, Pesce AJ. Reference Intervals: A User’s Guide. Washington DAP, editor: Washington, DC: AACC Press; 2005.

[35] Horowitz GL. Defining, Establishing, and Verifying Reference Intervals in the Clinical Laboratory, Approved Guideline. Defining, Establishing, and Verifying Reference Intervals in the Clinical Laboratory. 282010. doi: 10.7754/clin.lab.2015.150609 27012043

[36] Dowiasch S, Marx S, Einhauser W, Bremmer F. Effects of aging on eye movements in the real world. Front Hum Neurosci. 2015;9:46. Epub 2015/02/26. doi: 10.3389/fnhum.2015.00046 ; PubMed Central PMCID: PMC4322726. 25713524 PMC4322726

[37] Irving EL, Steinbach MJ, Lillakas L, Babu RJ, Hutchings N. Horizontal saccade dynamics across the human life span. Invest Ophthalmol Vis Sci. 2006;47(6):2478–84. Epub 2006/05/26. doi: 10.1167/iovs.05-1311 . 16723459

[38] Maruta J, Spielman LA, Rajashekar U, Ghajar J. Visual Tracking in Development and Aging. Front Neurol. 2017;8:640. Epub 2017/12/19. doi: 10.3389/fneur.2017.00640 ; PubMed Central PMCID: PMC5714854. 29250026 PMC5714854

[39] Kanayama R, Nakamura T, Sano R, Ohki M, Okuyama T, Kimura Y, et al. Effect of aging on smooth pursuit eye movement. Acta Otolaryngol Suppl. 1994;511:131–4. Epub 1994/01/01. doi: 10.3109/00016489409128316 8203214

[40] Fukata K, Amimoto K, Fujino Y, Inoue M, Inoue M, Takahashi Y, et al. The effects of aging on the subjective vertical in the frontal plane in healthy adults. J Phys Ther Sci. 2017;29(11):1950–3. Epub 2017/12/05. doi: 10.1589/jpts.29.1950 ; PubMed Central PMCID: PMC5702821. 29200631 PMC5702821

[41] Valmaggia C, Rutsche A, Baumann A, Pieh C, Bellaiche Shavit Y, Proudlock F, et al. Age related change of optokinetic nystagmus in healthy subjects: a study from infancy to senescence. Br J Ophthalmol. 2004;88(12):1577–81. Epub 2004/11/19. doi: 10.1136/bjo.2004.044222 ; PubMed Central PMCID: PMC1772432. 15548816 PMC1772432

[42] Luna B, Velanova K, Geier CF. Development of eye-movement control. Brain Cogn. 2008;68(3):293–308. doi: 10.1016/j.bandc.2008.08.019 ; PubMed Central PMCID: PMC2731686. 18938009 PMC2731686

[43] Hardin AP, Hackell JM, Committee On P, Ambulatory M. Age Limit of Pediatrics. Pediatrics. 2017;140(3). doi: 10.1542/peds.2017-2151 . 28827380

[44] Kelly KM, Kiderman A, Akhavan S, Quigley MR, Snell ED, Happ E, et al. Oculomotor, Vestibular, and Reaction Time Effects of Sports-Related Concussion: Video-Oculography in Assessing Sports-Related Concussion. The Journal of head trauma rehabilitation. 2018. Epub 2018/09/21. doi: 10.1097/HTR.0000000000000437 . 30234848 PMC6553977

[45] Maruta J, Ghajar J. Detecting eye movement abnormalities from concussion. Prog Neurol Surg. 2014;28:226–33. Epub 2014/06/14. doi: 10.1159/000358786 . 24923406

[46] Warden DL, Bleiberg J, Cameron KL, Ecklund J, Walter J, Sparling MB, et al. Persistent prolongation of simple reaction time in sports concussion. Neurology. 2001;57(3):524–6. Epub 2001/08/15. doi: 10.1212/wnl.57.3.524 . 11502926

[47] Hultsch DF, MacDonald SW, Dixon RA. Variability in reaction time performance of younger and older adults. J Gerontol B Psychol Sci Soc Sci. 2002;57(2):P101–15. Epub 2002/02/28. doi: 10.1093/geronb/57.2.p101 . 11867658

[48] Kansaku K, Hanakawa T, Wu T, Hallett M. A shared neural network for simple reaction time. Neuroimage. 2004;22(2):904–11. doi: 10.1016/j.neuroimage.2004.02.006 . 15193621

[49] Condy C, Rivaud-Pechoux S, Ostendorf F, Ploner CJ, Gaymard B. Neural substrate of antisaccades: role of subcortical structures. Neurology. 2004;63(9):1571–8. doi: 10.1212/01.wnl.0000142990.44979.5a . 15534238

[50] Kapoor N, Ciuffreda KJ. Vision Disturbances Following Traumatic Brain Injury. Curr Treat Options Neurol. 2002;4(4):271–80. Epub 2002/05/31. doi: 10.1007/s11940-002-0027-z . 12036500

[51] Ashmore RC, Sommer MA. Delay activity of saccade-related neurons in the caudal dentate nucleus of the macaque cerebellum. J Neurophysiol. 2013;109(8):2129–44. doi: 10.1152/jn.00906.2011 ; PubMed Central PMCID: PMC3628037. 23365182 PMC3628037

[52] Capo-Aponte JE, Urosevich TG, Temme LA, Tarbett AK, Sanghera NK. Visual dysfunctions and symptoms during the subacute stage of blast-induced mild traumatic brain injury. Mil Med. 2012;177(7):804–13. Epub 2012/07/20. doi: 10.7205/milmed-d-12-00061 . 22808887

[53] Samadani U. A new tool for monitoring brain function: eye tracking goes beyond assessing attention to measuring central nervous system physiology. Neural Regen Res. 2015;10(8):1231–3. Epub 2015/10/22. doi: 10.4103/1673-5374.162752 ; PubMed Central PMCID: PMC4590232. 26487847 PMC4590232

[54] Cochrane GD, Gould SJ, Sheehan N, Busettini C, Christy JB, Weise KK, et al. Saccadic intrusions in paediatric concussion. Clinical & experimental optometry. 2020. Epub 2020/01/24. doi: 10.1111/cxo.13045 . 31970807 PMC8034559

