# Supplementary material for: Portable eye-tracking as a reliable assessment of oculomotor, cognitive and reaction time function: Normative data for 18–45 year old
Source: PLoS One. 2021 Nov 22;16(11):e0260351. doi: 10.1371/journal.pone.0260351 (PMC8608311; doi:10.1371/journal.pone.0260351)
Supplement: S1 Table — (PDF) [file pone.0260351.s001.pdf]

S1 Table. Inclusion/exclusion criteria.

**Inclusion criteria**
• Males or females from 18 – 45 years of age and of all races<br>The research team has chosen a cutoff of 45 years of age in order to avoid introducing wide variance in the oculomotor, vestibular and reaction time results for the control group. The team believes that older people (above 45 years of age) are likely to exhibit a much wider range of responses as a natural consequence of aging.
**Exclusion criteria**
• Brain injury resulting from a penetrating wound to the head, neck, face or brain (to include gunshot wounds)<br>• Presence of severe aphasia*<br>• Persons whom have had an mTBI within the prior month and are symptomatic.<br>• Persons with a previous history of multiple mTBIs and are symptomatic.<br>• Concussion within the last year (12 months)<br>• Repeated blast exposure*<br>• History of neuropsychiatric disorders antedating the head injury (e.g. hypochondriasis, major depression, schizophrenia)<br>• Pregnancy<br>• Prior disorders of hearing and balance including:<br>• Meniere's disease<br>• Chronic migraine<br>• Multiple sclerosis

- Vestibular neuritis
    - Vestibular schwannoma
    - Sudden sensorineural hearing loss
- Cerebrovascular disorders
- Systemic disorders: e.g. chronic renal failure, cirrhosis of the liver, etc.
- Medications which depress the sensorium precluding patient compliance with the testing (considered on a case-by-case basis)
- Previous contraindicating surgeries at the discretion of the study physicians or audiologists
