# Supplementary material for: Portable eye-tracking as a reliable assessment of oculomotor, cognitive and reaction time function: Normative data for 18–45 year old
Source: PLoS One. 2021 Nov 22;16(11):e0260351. doi: 10.1371/journal.pone.0260351 (PMC8608311; doi:10.1371/journal.pone.0260351)
Supplement: S2 Table — Data present the 2.5, 5, 10, 25, 75, 90, 95 and 97.5 percentile for each metric. (PDF) [file pone.0260351.s002.pdf]

S2 Table. Percentiles for saccades and smooth pursuit tests. Data present the 2.5, 5, 10, 25, 75, 90, 95 and 97.5 percentile for each metric.

Test: Saccade – Random, Horizontal					
		Latency (s)	Accuracy (%)	Final accuracy (%)	AUF (deg^2/s)
Percentiles	2.5	0.14	81	88	8205
	5	0.14	83	90	8591
	10	0.15	85	92	9141
	25	0.16	89	94	9810
	75	0.19	96	98	11054
	90	0.20	98	100	11633
	95	0.21	100	102	12015
	97.5	0.22	103	104	12247
Test: Saccade – Random, Vertical					
		Latency (s)	Accuracy (%)	Final accuracy (%)	AUF (deg^2/s)
Percentiles	2.5	0.14	75	79	7630
	5	0.15	77	81	7903
	10	0.16	80	85	8253
	25	0.17	87	90	9054
	75	0.20	99	100	10791
	90	0.21	104	103	11434
	95	0.22	107	105	11818
	97.5	0.23	110	107	12025

Test: Smooth Pursuit – Horizontal 0.1Hz: Percentiles					
		Velocity gain	Asymmetry (%)	Position gain	Saccadic component (%)
Percentiles	2.5	0.78	-8.87	0.96	3.04
	5	0.82	-8.07	0.96	4.04
	10	0.85	-6.55	0.98	5.94
	25	0.91	-3.13	0.99	9.85
	75	1.01	2.04	1.02	22.22
	90	1.04	5.22	1.03	29.12
	95	1.05	6.89	1.03	33.33
	97.5	1.07	7.59	1.04	35.40
Test: Smooth Pursuit – Horizontal 0.75Hz					
		Velocity gain	Asymmetry (%)	Position gain	Saccadic component (%)
Percentiles	2.5	0.62	-9.51	0.79	2.30
	5	0.75	-5.19	0.83	2.94
	10	0.83	-3.37	0.88	4.73
	25	0.92	-1.48	0.94	8.07
	75	1.02	1.77	1.02	20.57
	90	1.05	4.40	1.07	30.09
	95	1.08	6.72	1.09	33.16
	97.5	1.08	9.15	1.10	36.59
Test: Smooth Pursuit – Vertical 0.1Hz					

		Velocity gain	Asymmetry (%)	Position gain	Saccadic component (%)
Percentiles	2.5	0.69	-12.67	0.95	2.39
	5	0.72	-11.35	0.96	3.88
	10	0.77	-9.33	0.96	5.56
	25	0.84	-5.83	0.96	8.16
	75	0.97	3.38	1.02	18.56
	90	1.02	7.60	1.04	25.02
	95	1.05	9.41	1.05	27.84
	97.5	1.07	11.51	1.06	31.24
Test: Smooth Pursuit – Vertical 0.75Hz					
		Velocity gain	Asymmetry (%)	Position gain	Saccadic component (%)
Percentiles	2.5	0.41	-23.60	0.73	3.89
	5	0.45	-20.13	0.76	7.04
	10	0.56	-11.68	0.80	9.99
	25	0.71	-3.46	0.86	16.66
	75	0.95	10.20	1.01	36.97
	90	1.02	20.01	1.06	45.26
	95	1.06	24.90	1.09	48.96
	97.5	1.09	29.07	1.11	51.87
