# Supplementary material for: Portable eye-tracking as a reliable assessment of oculomotor, cognitive and reaction time function: Normative data for 18–45 year old
Source: PLoS One. 2021 Nov 22;16(11):e0260351. doi: 10.1371/journal.pone.0260351 (PMC8608311; doi:10.1371/journal.pone.0260351)
Supplement: S3 Table — Data represent the 2.5, 5, 10, 25, 75, 90, 95 and 97.5 percentile for each metric. (PDF) [file pone.0260351.s003.pdf]

S3 Table. Percentiles for predictive saccades and antisaccades. Data represent the 2.5, 5, 10, 25, 75, 90, 95 and 97.5 percentile for each metric.

Test: Predictive Saccade				
		Percentage predicted (%)	18-21 Percentage predicted (%)	22-45 Percentage predicted (%)
Percentiles	2.5	16.67	15.91	17.39
	5	24.11	16.67	26.09
	10	29.50	25.00	34.78
	25	45.99	36.36	52.17
	75	78.26	73.32	82.61
	90	86.96	82.61	91.30
	95	91.30	86.96	95.45
	97.5	95.62	90.12	95.65
Test: Antisaccades				
		Error rate (%)		
Percentiles	2.5	0		
	5	0		
	10	0		
	25	6.25		
	75	25		
	90	37.5		
	95	46.6		

	97.5	50
