# Supplementary material for: Portable eye-tracking as a reliable assessment of oculomotor, cognitive and reaction time function: Normative data for 18–45 year old
Source: PLoS One. 2021 Nov 22;16(11):e0260351. doi: 10.1371/journal.pone.0260351 (PMC8608311; doi:10.1371/journal.pone.0260351)
Supplement: S4 Table — Data represent the 2.5, 5, 10, 25, 75, 90, 95 and 97.5 percentile for each metric. (PDF) [file pone.0260351.s004.pdf]

S4 Table. Percentiles for reaction time tests. Data represent the 2.5, 5, 10, 25, 75, 90, 95 and 97.5 percentile for each metric.

Test: Visual Reaction Time		
		Latency (ms)
Percentiles	2.5	220.12
	5	228.58
	10	235.00
	25	247.08
	75	287.50
	90	311.80
	95	332.55
	97.5	342.50

Test: Auditory Reaction Time				
		Latency (ms) (combined male and female data)	Female - Latency (ms)	Male - Latency (ms)
Percentiles	2.5	163.33	161.10	161.33
	5	168.81	176.94	167.78
	7.5	174.65	186.90	172.15
	10	178.89	192.14	175.00
	25	195.56	206.67	192.14
	75	247.84	265.00	238.33

	90	281.11	300.00	268.67
	95	304.16	321.07	295.00
	97.5	317.71	336.13	305.34
Test: Saccade and Reaction Time				
Saccade variables:		Latency (s)	Accuracy (%)	Final accuracy (%)
Percentiles	2.5	0.14	73.64	78.41
	5	0.15	76.60	83.38
	10	0.16	81.01	86.83
	25	0.17	84.93	91.10
	75	0.21	92.87	97.03
	90	0.24	95.71	99.57
	95	0.27	98.26	101.98
	97.5	0.29	100.78	105.86
Motor variables		Latency mean (s) – for Left Button	Latency mean (s) – for Right Button	
Percentiles	2.5	0.25	0.24	
	5	0.26	0.26	
	10	0.29	0.28	
	25	0.35	0.34	
	75	0.48	0.46	
	90	0.57	0.54	
	95	0.62	0.58	

	97.5	0.66	0.65
