# Supplementary material for: Portable eye-tracking as a reliable assessment of oculomotor, cognitive and reaction time function: Normative data for 18–45 year old
Source: PLoS One. 2021 Nov 22;16(11):e0260351. doi: 10.1371/journal.pone.0260351 (PMC8608311; doi:10.1371/journal.pone.0260351)
Supplement: S5 Table — Mean and standard deviation (SD) is presented for each test metric. (PDF) [file pone.0260351.s005.pdf]

S5 Table. Descriptive statistics. Mean and standard deviation (SD) for each test metric.

Test	Metrics	Mean	SD
Saccade – Random, Horizontal	Latency (s)	0.17	0.02
	Accuracy (%)	92.11	5.12
	Final accuracy (%)	96.27	4.23
	AUF (deg^2/s)	10397	999
Saccade – Random, Vertical	Latency (s)	0.18	0.02
	Accuracy (%)	92.59	8.93
	Final accuracy (%)	94.45	7.11
	AUF (deg^2/s)	9887	1197
Smooth Pursuit – Horizontal 0.1Hz	Velocity gain	0.95	0.07
	Asymmetry (%)	-0.53	4.24
	Position gain	1.00	0.02
	Saccadic component (%)	16.51	8.57
Smooth Pursuit – Horizontal 0.75 Hz	Velocity gain	0.95	0.10
	Asymmetry (%)	0.12	3.98
	Position gain	0.98	0.07
	Saccadic component (%)	14.90	9.30
Smooth Pursuit – Vertical 0.1 Hz	Velocity gain	0.90	0.10
	Asymmetry (%)	-1.11	6.36
	Position gain	1.00	0.03
	Saccadic component (%)	13.97	7.44

Smooth Pursuit – Vertical 0.75 Hz	Velocity gain	0.82	0.17
	Asymmetry (%)	3.11	12.49
	Position gain	0.94	0.10
	Saccadic component (%)	27.07	13.07
Predictive Saccades	Percentage predicted (%)	61.84	21.84
Antisaccades	Error rate (%)	17.97	13.78
Visual Reaction Time	Latency (ms)	270.2	30.1
Auditory Reaction Time	Latency (ms)	224.3	39.7
Saccade and Reaction Time	Latency (s)	0.20	0.04
